# Clinical Diagnosis and Treatment of Gingival Metal Tattoo Using Optical Coherence Tomography and Er:YAG Laser-Assisted Microsurgery: A Case Series with an Ex Vivo Proof-of-Concept Study

**DOI:** 10.3390/dj14100680

**Published:** 2026-10-19

**Authors:** Risako Mikami, Masaki Tsubokawa, Koji Mizutani, Taichen Lin, Yoichi Taniguchi, Verica Pavlic, Takanori Iwata, Yasunori Sumi, Akira Aoki

**Affiliations:** 1 Department of Advanced Biomaterials, Graduate School of Medicine and Dentistry, Institute of Science Tokyo, Tokyo 113-8549, Japan; 2 Department of Periodontology, Graduate School of Medicine and Dentistry, Institute of Science Tokyo, Tokyo 113-8549, Japan; tsubokawa.dental@gmail.com (M.T.);; 3 Tsubokawa Dental Clinic, Samukawa 253-0105, Japan; 4 Department of General Dentistry, Graduate School of Medicine and Dentistry, Institute of Science Tokyo, Tokyo 113-8510, Japan; 5 Department of Dentistry, Chung Shan Medical University Hospital, Taichung 40201, Taiwan; 6 School of Dentistry, Chung Shan Medical University, Taichung 40201, Taiwan; 7 Taniguchi Dental Clinic, Sapporo 003-0023, Japan; 8 Department of Periodontology and Oral Medicine, Institute of Dentistry, 78000 Banja Luka, Bosnia and Herzegovina; 9 Department of Periodontology and Oral Medicine, Medical Faculty University of Banja Luka, 78000 Banja Luka, Bosnia and Herzegovina; 10 Department of Cariology and Operative Dentistry, Graduate School of Medical and Dental Sciences, Institute of Science Tokyo, Tokyo 113-8549, Japan

**Keywords:** gingival metal tattoo, gingival pigmentation, optical coherence tomography, Er:YAG laser, microsurgery, esthetic dentistry, non-invasive diagnosis

## Abstract

**Background**: Gingival metal tattoos caused by prosthodontic treatment present esthetic problems. To date, their removal has been unpredictable because of invasive surgical methods and the lack of reliable diagnostic tools. Objective: This study investigated the potential utility of optical coherence tomography (OCT) for the non-invasive assessment of metal tattoos and evaluated the minimally invasive removal using Er:YAG laser microsurgery. **Methods**: This study comprised an exploratory ex vivo proof-of-concept experiment using three gingival metal tattoo models and a clinical case series of three patients with gingival metal tattoos. Ex vivo models were created using porcine gingiva with artificially embedded metal fragments, which were analyzed through OCT and histological examination. In the clinical cases, three women with anterior gingival metal tattoos underwent OCT followed by Er:YAG laser microsurgery under microscopic guidance between 2012 and 2013. **Results**: Ex vivo experiments showed highly reflective OCT signals corresponding to the histological locations of metal fragments within the connective tissue. Clinically, OCT enabled visualization and localization of findings consistent with metal fragments and provided guidance for Er:YAG laser removal. All three patients showed uneventful healing without postoperative pain. Follow-up was available for 1.5 months in Case 1 and for up to 13 years in Cases 2 and 3, with favorable long-term clinical and esthetic outcomes observed in the latter two cases. **Conclusions**: OCT may facilitate non-invasive assessment and localization of gingival metal tattoos. When combined with Er:YAG laser microsurgery, it may provide a feasible and minimally invasive approach with favorable esthetic outcomes.

## 1. Introduction

Gingival metal tattoos caused by metal fragments that were ground intraorally during prosthodontic treatment and invaded surrounding gingiva evoke an esthetic concern, especially in gingival tissue of the anterior teeth. In order to remove metal tattoos, invasive surgeries such as connective tissue graft technique [1,2,3] or free gingival grafting [4] following gingivectomy have been proposed, although there are still no established effective methods. A recent literature review also identified graft-based procedures and laser-based approaches as the principal treatment modalities reported for esthetic management of amalgam tattoos [5]. Currently, lasers are considered to be useful for treatment of oral soft tissues [6]. We have previously proposed an effective and safe microsurgery for metal tattoo removal using the Er:YAG laser [7] and also established its specific technical modality of Er:YAG laser micro-keyhole surgery (EL-MIKS) [8,9].

Metal tattoos are the pigmentation of gingival connective tissue derived from metal debris that have strayed into the gingival connective tissue as well as its gradual dissolution. The success of the treatment procedure depends on the precise removal of these metal debris. Similar to metal tattoos, melanin pigmentation is a cause of the gingival esthetic problem [10]. In gingival melanin pigmentation, melanin is located mainly in the basal and suprabasal cell layers of the oral epithelium [11], whereas metal debris of metal tattoos are deposited in the connective tissue generally, making their removal more invasive and less predictable than melanin depigmentation. Regarding the diagnosis, there have been no non-invasive methods for detecting the presence and location of metal debris and diagnosing the metal tattoo, and therefore the diagnosis was performed only by visual observation based on clinical experiences as well as medical interview, although occasionally dental radiographs slightly reveal the presence of some metal debris. Moreover, the removal of metal tattoos has only been performed by searching for metal micro-fragments based on clinical estimation of the extent and degree of pigmentation. This was another factor that made the metal tattoo removal procedure less predictable.

Optical coherence tomography (OCT) is a high-resolution, non-invasive optical imaging technique for creating cross-sectional images of hard and soft tissues [12]. OCT apparatus irradiates the object with the near-infrared light and performs a fast Fourier transform of the reflected backscattered light to produce a tomographic image of biological tissue in real time. OCT has been applied in dentistry [13], which enables the observation of periodontal tissues and the detection of dental caries and subgingival calculus in real time [14,15]. More recently, OCT applications in periodontology have expanded to three-dimensional imaging and OCT angiography, allowing detailed assessment of periodontal morphology and microvascular changes [16,17,18]. Recent advances in portable OCT systems and image analysis technologies may further broaden its potential clinical applications in periodontal diagnosis and monitoring [16,17,18]. OCT is a light-based imaging device and its image resolution is higher than that of other biological tomographic imaging devices such as computed tomography (CT) and ultrasound tomography. OCT has another advantage of no radiation exposure.

OCT is an apparatus that relies on light transparency. As light transmission differs significantly between biological tissues and metal debris, metal fragments causing the metal tattoo may be easily delineated and located with OCT. Also, when OCT accurately locates the metal fragments in the connective tissue, careful Er:YAG laser irradiation under microscopic monitoring enables more precise and minimally invasive removal. In this report, first, an ex vivo model of periodontal tissue with a metal tattoo was used as a proof of concept to examine whether metal fragments embedded in gingival connective tissue could be visualized by OCT and whether the OCT findings corresponded to their histological location. Subsequently, a clinical application of OCT combined with Er:YAG laser microsurgery was evaluated in a case series.

## 2. Materials and Methods

### 2.1. OCT System and Imaging Protocol

The OCT systems (Inner Vision IV-2000, Santec, Komaki, Japan; and Prototype 2, Panasonic Health Care, Toon, Japan) employing frequency (Fourier) domain techniques with a tunable light source were used. The center wavelengths and bandwidths of the scanning lasers were 1310 nm/100 nm for IV-2000, and 1330 nm/100 nm for Prototype 2. The IV-2000 system consists of a high-speed scanning frequency-swept external cavity laser and interferometer unit, a scanning probe, and a personal computer. The illumination power of the sample arm is 0.8 mW, and the power of the reference arm is 0.6 μW. A balance detector is utilized for interference detection, and a data acquisition card is used for computer/photodetector interfacing. The system incorporates a hand-held probe to set appropriate distances from the sites. The output power is <5 mW, which is within the safety limit for class I laser instruments as defined by the American National Standards Institute. The electric signal acquisition rate is 100 MS/s. The light beam from the laser source projected onto the area of interest was scanned using the hand-held probe. Backscattered light relaying information about the microstructure of the tissue was collected, returned to the system, digitized on a time-scale, and analyzed in the Fourier domain to reveal the depth of the area of interest. In this case series, tomographic imaging of periodontal tissue profile of the pigmented area was performed by OCT immediately before the metal tattoo removal procedure and after complete wound healing.

### 2.2. Ex Vivo Study

#### 2.2.1. Biological Materials and Preparation of Artificial Metal Tattoo Models

This exploratory ex vivo experiment was conducted as a proof of concept to examine the visualization of metal fragments in gingival connective tissue by OCT and their correspondence with histological findings. It was not designed to assess the diagnostic accuracy of OCT; therefore, no formal sample size calculation was performed. Human extracted teeth collected with the consent of the volunteer patients were employed (ethics committee approval D2014-131, The Research Ethics Committee of Faculty of Dentistry, Tokyo Medical and Dental University (TMDU), currently Institute of Science Tokyo (Science Tokyo)). The teeth were extracted because of severe periodontitis. After extraction, remaining soft tissue on the root surface was removed by 30 min of ultrasonic cleaning and immersion in 5% sodium hypochlorite. The teeth were stored in water at 4 °C until use.

Porcine gingival samples obtained from the mandibles of pigs slaughtered for food production were used as experimental models for human oral mucosa [19]. Full-thickness porcine keratinized gingival sheets, 20 mm × 7 mm, were prepared from porcine mandibles. A gold/silver/palladium alloy was ground with a dental rotary instrument and the collected metal fragments were used in this experiment. Several linear small incisions were made randomly with a scalpel on the periosteal side of the porcine gingival sheets into the connective tissue, and the metal fragments were inserted into the connective tissue along the linear incisions to create artificial metal tattoos. The porcine gingival sheets with metal fragments were placed on the root surfaces of human extracted teeth. Three sets of these artificial metal tattoo models were prepared.

#### 2.2.2. OCT Imaging

Photographs and OCT tomographic images were taken before and after insertion of the metal fragments. In order to take OCT images, each tooth–gingival sheet model was fixed on a three-dimensional translation stage, and an OCT image parallel to the apico-coronal axis of the tooth was obtained at the center of the gingival sheet, including the artificial tattoo site.

#### 2.2.3. Histological Analysis

For histological analyses, the gingival sheet samples were fixed in 10% formalin solution, followed by graded ethanol solutions, and defatted in acetone, cleared with monomer, and then embedded in methyl methacrylate resin for preparation of the non-decalcified specimens. After 3 weeks of polymerization at 35 °C, the resin blocks were trimmed and cut. For each gingival sheet sample, one longitudinal section parallel to the long axis of the sample was carefully prepared with a micro-cutting machine (BS-3000, EXAKT, Norderstedt, Germany). The thickness of the section was then reduced to approximately 20–25 μm via polishing with a micro-grinding machine (MG-400, EXAKT). The sections were stained with toluidine blue and examined using a light microscope (E800, Nikon Inc., Tokyo, Japan).

### 2.3. Clinical Cases

#### 2.3.1. Patients and Settings

The clinical component comprised a case series of three patients and was exploratory in nature. The clinical case series was reported in accordance with the applicable items of the CARE (Case REport) guidelines [20]. This case series included the patients who visited the Periodontology Department of TMDU Hospital (currently Science Tokyo Hospital) between 2012 and 2013. The main complaints of the three patients were unesthetic gingival pigmentation related to prosthodontic treatment of anterior teeth. The three patients were women aged 32, 34, and 48 years, and none of them had a history of smoking.

All three patients had clinically healthy gingiva except for metal tattoos. Tooth brushing instruction and professional tooth cleaning were performed prior to metal tattoo removal. Informed consent was obtained from each patient before OCT examination and Er:YAG laser treatment. Informed consent was obtained from each patient before the OCT examination, including consent for the use of the examination results for research purposes and academic presentation. The retrospective use of clinical data from the patients’ medical records was conducted under an opt-out procedure approved by the Research Ethics Committee of Faculty of Dentistry, TMDU (currently Science Tokyo) (Approval numbers: 527 and D2020-20).

#### 2.3.2. Clinical OCT Examination

After obtaining the patients’ complete medical histories, clinical examinations, including OCT imaging, were performed. The OCT images were qualitatively evaluated for the presence, distribution, and location of highly reflective signals within the gingival connective tissue that were considered to correspond to metal fragments. Post-treatment OCT images were compared qualitatively with the preoperative images to assess changes in these reflective signals. OCT images were interpreted by an experienced periodontist (A.A.) as part of the clinical assessment, and no independent blinded evaluation was performed.

#### 2.3.3. Er:YAG Laser Treatment

Metal tattoo removal was performed according to our previous reports [7,8,9]. Briefly, the metal debris and the surrounding discolored connective tissue were carefully removed with the Er:YAG laser (DELight Dental Laser; Hoya ConBio, Fremont, CA, USA) under the 10–20× magnified view with a surgical microscope (OPMI 111, Carl Zeiss, Oberkochen, Germany). Vertical or oblique contact irradiation was performed under a water spray using a curved contact tip with a tip dimeter of 600 μm. The papillary edges and free gingival margins were left untreated to prevent detachment of marginal gingival tissue and subsequent gingival recession. In this case series, metal tattoo removal was performed according to the concept of EL-MIKS [8]. In EL-MIKS, a micro-keyhole with a diameter of 1–3 mm is prepared at a suitable site in the discolored area as a starting point for laser ablation. The accessible metal pieces and surrounding pigmented connective tissue were then carefully evaporated as much as possible at the under-cut area through the micro-keyhole, by changing the irradiation direction within the inside of the hole. Depending on pigmentation, one or a few micro-keyholes with a suitable distance from each other were prepared for each session. After the space created by the tissue evaporation was filled with blood clot, patients were instructed to cease tooth brushing around the treated sites for 4–5 days. No antibiotics or analgesics were prescribed. Depending on cases, the procedure was divided into two to three sessions on different days at intervals of 1 month in order to secure the complete healing of the treated sites. Postoperative pain from the immediate postoperative period to seven days was assessed using the visual analog scale (VAS). Esthetic outcomes were assessed qualitatively based on clinical examination and photographs. All the treatments and assessments were performed by a single experienced periodontist (A.A.).

## 3. Results

### 3.1. Ex Vivo Experiments

**Figure 1:**
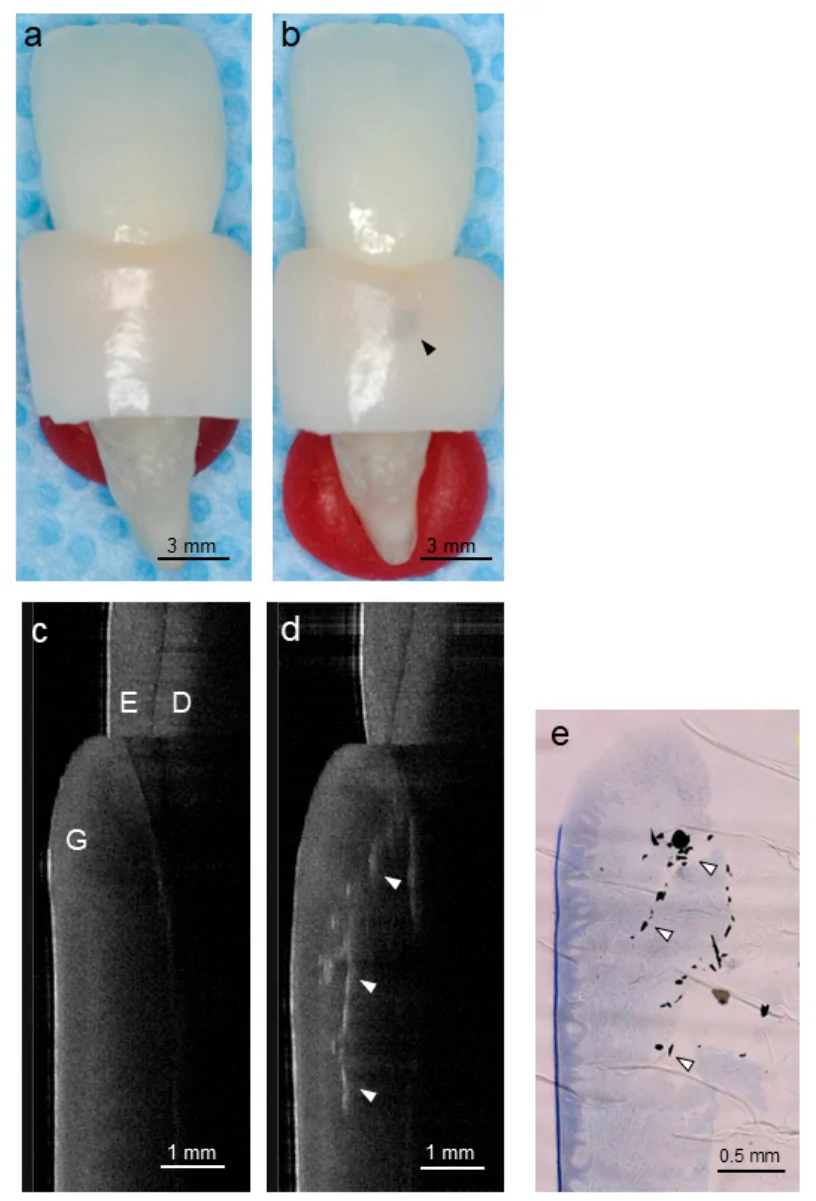
A representative case of ex vivo experiments. (**a**) Porcine gingival model with human extracted teeth prior to insertion of metal fragments into the gingival connective tissue. (**b**) After insertion of metal fragments. Arrowhead indicates a black area where the presence of metal fragments is suggested through the gingival tissue. (**c**) OCT image before insertion of metal fragments. E: enamel, D: dentin, G: gingival tissue. (**d**) OCT image after insertion of metal fragments. Arrowheads indicate white dots and lines considered to be reflection from metal fragments in the gingival tissue. (**e**) Histological image stained with toluidine blue. Arrowheads indicate metal fragments in connective tissue.

In the experimental artificial tattoo model created in this study, metal fragments inserted into the connective tissue of gingival sheets were externally visible as a black mass, similar to the appearance of clinical metal tattoos (Figure 1b). In the OCT image before insertion of the metal fragments, the internal structure of the gingiva could be confirmed (Figure 1c). Compared with the images before metal fragment insertion, the OCT images of the artificial tattoo model with metal fragments revealed strong white reflections consistent with the areas where metal fragments could be seen on the histological images (Figure 1d,e), indicating correspondence between the OCT findings and the histological location of the metal fragments (Figure 1d). These findings were obtained in all three samples.

### 3.2. Clinical Cases

The clinical characteristics, OCT findings, treatment procedures, and outcomes of the three cases are summarized in Table 1.

#### 3.2.1. Case 1

**Figure 2:**
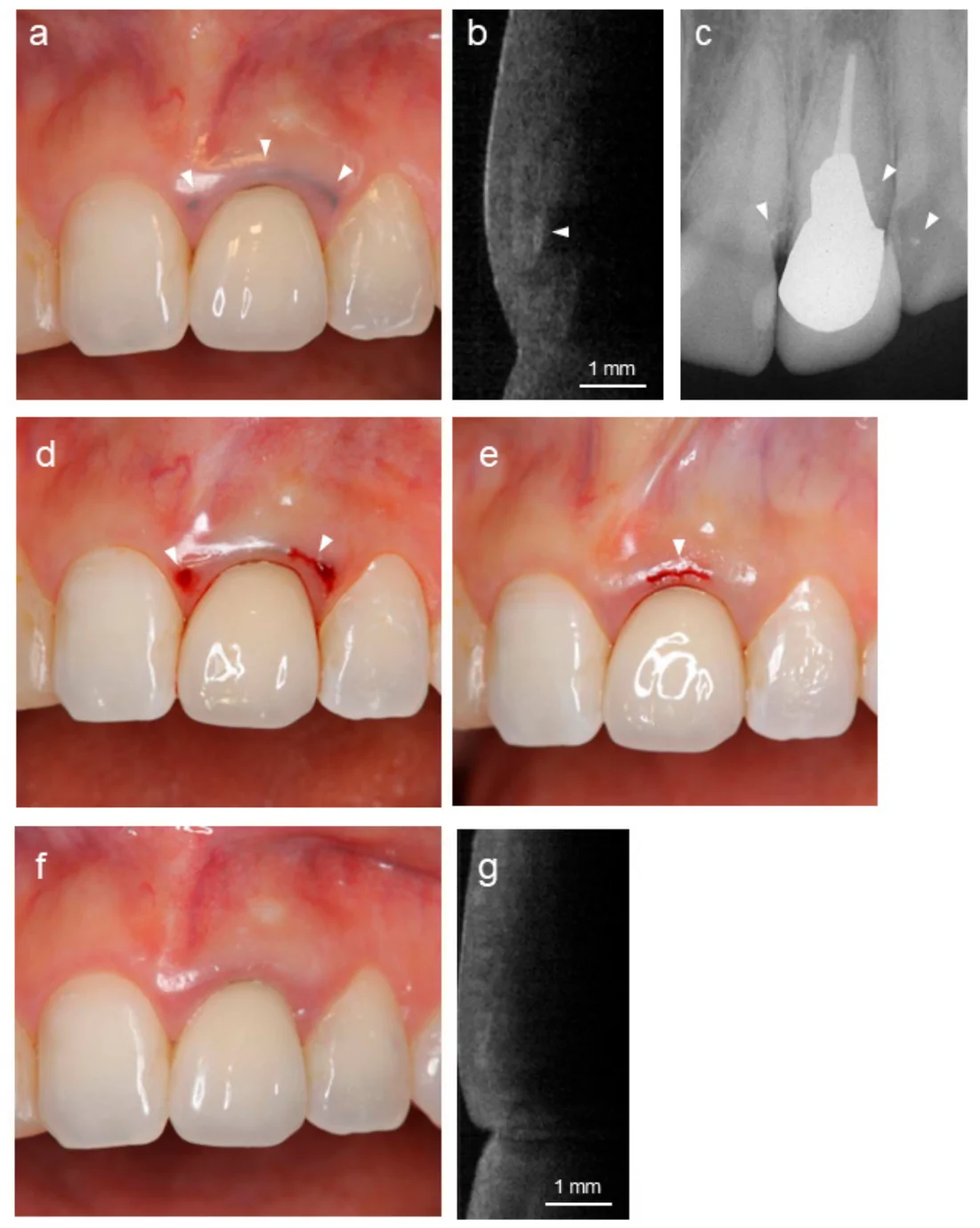
Case 1. A 32-year-old female with (**a**) linear pigmentation along the gingival margin of the left upper central incisor (arrowheads). (**b**) Preoperative OCT image at the center of the tooth. A small white area in the connective tissue is observed (arrowhead). (**c**) Preoperative radiograph. Small white dots are slightly visible (arrowheads). (**d**) Metal tattoo removal was performed in two separate appointments to avoid gingival recession due to postoperative reduced blood flow in the remaining thin marginal gingiva. In the first appointment, the mesial and distal sites (arrowheads) were treated by two minimal round and linear access holes. (**e**) At 20 days after the first appointment, the mesial and distal sites were depigmented well. The central area (arrowhead) was treated in the second appointment. (**f**) Intraoral photograph one and a half months postoperatively. Successful removal of discoloration has been achieved, and the gingiva has a healthy pink color. (**g**) Postoperative OCT image. The white area observed preoperatively has disappeared.

A 32-year-old woman. A narrow band of black pigmentation on the marginal gingiva of the left maxillary central incisor was observed (Figure 2a). From the medical interview, she had had a porcelain-fused metal crown placed several years ago and had been aware of the pigmentation after the treatment. Preoperative OCT imaging revealed a white signal area with slightly indistinct borders in the gray area of connective tissue, suggesting the presence of metal debris (Figure 2b). A dental radiograph also slightly detected white spots (Figure 2c). Based on the intraoral examination, the medical history, the radiograph, and the OCT examination, a diagnosis of metal tattoo incidental to the prosthodontic treatment of the left maxillary central incisor was made. The metal tattoo was extensively deposited near the marginal gingival margin and proximally, and was treated in two separate appointments, as removing it all at once would worsen marginal gingival blood circulation and increase the risk of gingival recession (Figure 2d,e). Er:YAG laser gingival depigmentation was performed using a power setting of 40–50 mJ/pulse and a frequency of 30 Hz, with water spray, under a surgical microscope. After each treatment session, the patient reported a VAS score of zero, i.e., no pain at all, for postoperative pain on that day and throughout the first seven postoperative days. At the one-and-a-half-month postoperative follow-up, the wound was completely epithelialized, and gingival coloration had improved without deformity or gingival retraction (Figure 2f). Postoperative OCT images showed that the white areas observed preoperatively had disappeared, suggesting that the metal fragments had been removed (Figure 2g). In this case, long-term follow-up was not available since the patient was an overseas resident and left Japan shortly after completion of the treatment.

#### 3.2.2. Case 2

**Figure 3:**
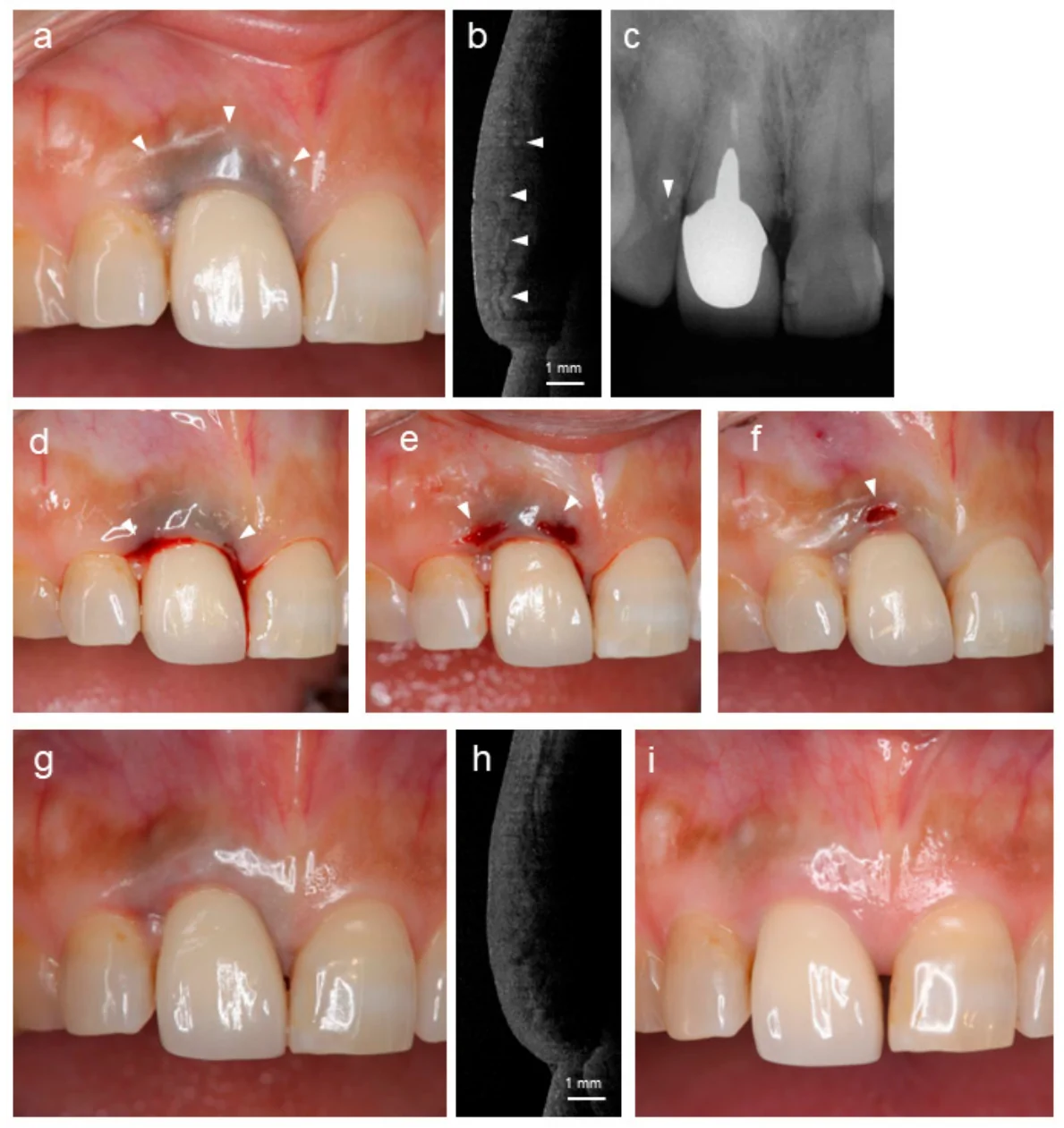
Case 2. A 48-year-old female. (**a**) Severe broad pigmentation (arrowheads) was observed in the entire gingiva of the right upper central incisor. (**b**) Preoperative OCT image at the center of the tooth. Many diffuse white dots within the connective tissue are observed (arrowheads). (**c**) Preoperative radiograph. A white dot and a white small area were detected in the interdental site (arrowhead) but they were not certain. (**d**) Metal tattoo removal was performed in three separate appointments. In the first appointment, the mesial and distal sites (arrowheads) very close to the gingival margin were treated. (**e**) Three months after the first appointment, the mesial and distal sites slightly above the first treated sites (arrowheads) were treated in the second appointment. (**f**) Two months after the second session, discoloration of the mesial and distal sites became weaker. The central site (arrowhead) was treated in the third appointment. (**g**) Intraoral photograph 10 months postoperatively. Discoloration has been improved. Although slight discoloration remained, the patient was satisfied with the improvement of appearance and did not want any additional treatments. (**h**) Postoperative OCT image. The white dots observed preoperatively have disappeared. (**i**) Intraoral photograph taken 13 years after the final treatment session. Compared with the 10-month postoperative image (**g**), the gingival discoloration demonstrated significant improvement, showing healthy pink-colored gingiva and morphology, without any further intervention.

A 48-year-old woman. The chief complaint is discoloration of the gingiva of the right maxillary central incisor. Severe discoloration of the marginal gingiva in a broad area was observed (Figure 3a). Preoperative OCT imaging showed diffuse white signal areas scattered in the connective tissue, suggesting the presence of metal debris (Figure 3b). The tooth had a porcelain-fused metal crown, and a diagnosis of metal tattoo was made based on medical history, the intraoral observation, and the OCT observation. Due to the large extent of the metal tattoo in the entire gingiva, a removal procedure was performed in three individual appointments (Figure 3d–f). Er:YAG laser gingival depigmentation was performed using a power setting of 60 mJ/pulse and a frequency of 20 Hz, with water spray, under a surgical microscope. After each treatment session, the patient reported a VAS score of zero on that day and throughout the first seven postoperative days.

Due to the patient’s circumstances, the postoperative follow-up was conducted ten months after surgery, at which time the wound was completely epithelialized and gingival coloration improved without gingival deformity; however, there was a slight gingival recession and reduced pigmentation remained (Figure 3g). Additional depigmentation procedures were proposed; however, the patient was already satisfied with the improved appearance and did not want any additional treatments. OCT images did not show any spotty white areas in the connective tissue, as was observed preoperatively, suggesting that the metal debris had been removed (Figure 3h). At the 13-year follow-up, the gingival discoloration demonstrated significant improvement showing a healthy pink-colored marginal gingiva and morphology, with the patient satisfied with the esthetic outcome (Figure 3i).

#### 3.2.3. Case 3

**Figure 4:**
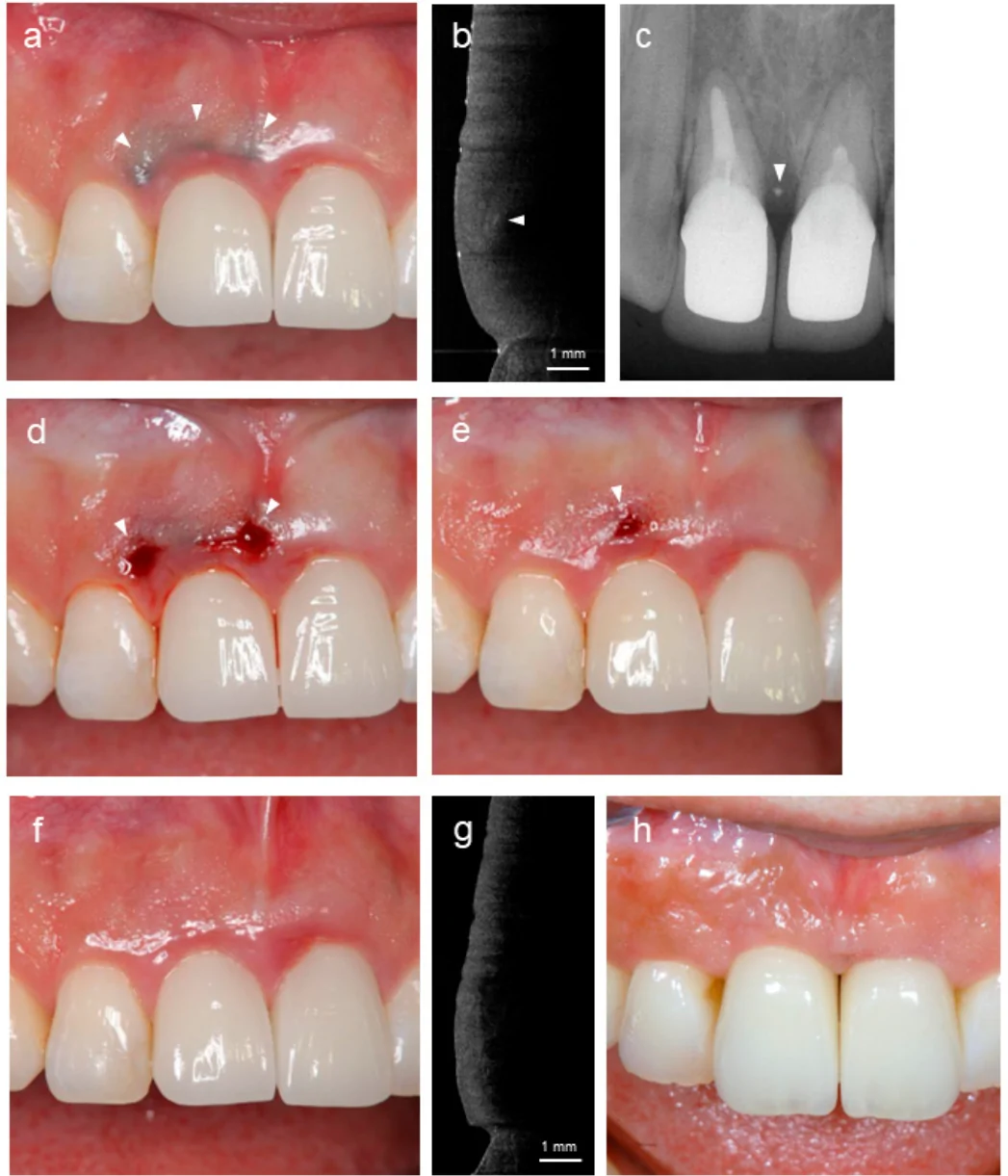
Case 3. A 34-year-old female. (**a**) A band of pigmentation along the gingival margin of the right upper central incisor (arrowheads). (**b**) Preoperative OCT image. White dots were observed in the connective tissue (arrowheads). (**c**) Preoperative radiograph. A white dot is visible (arrowhead). (**d**) Metal tattoo removal was performed in two separate appointments. In the first appointment, the mesial and distal sites were treated (arrowheads). (**e**) One and a half months after the first appointment, the mesial and distal sites were depigmented favorably. The central area was treated in the second appointment (arrowheads). (**f**) Intraoral photograph eight months postoperatively. Discoloration has been removed, and the gingiva has a healthy pink color. (**g**) Postoperative OCT image. The white dots observed preoperatively have disappeared. (**h**) Intraoral photograph obtained 13 years postoperatively. During follow-up, although extraction of both maxillary central incisors and placement of implants were performed in a private clinic because of root fractures, the peri-implant gingiva exhibited a stable, healthy appearance without recurrent pigmentation. ((**h**): Courtesy of Dr. Yoshiaki Hasegawa).

A 34-year-old woman. She visited the clinic because she was concerned about the gingival discoloration of her right maxillary central incisor. A dark discoloration band was observed on the marginal gingiva (Figure 4a). A ceramic crown had been placed on the tooth five months before the visit, and until this time she had previously had a porcelain-fused metal crown for 15 years. Preoperative OCT images showed well-defined white dots in the connective tissue, suggesting the presence of metal debris (Figure 4b). A dental radiograph also revealed a white dot at the interdental area. Based on her medical history, clinical observation, the radiograph, and the OCT analysis, the gingival pigmentation was diagnosed as a metal tattoo.

To avoid impaired wound healing and subsequent gingival recession, metal tattoo removal was performed in two separate sessions (Figure 4d,e). Er:YAG laser gingival depigmentation was performed using a power setting of 60 mJ/pulse and a frequency of 20 Hz, with water spray, under a surgical microscope. After each treatment session, the patient reported a VAS score of zero on that day and throughout the first seven postoperative days. Due to the patient’s circumstances, the postoperative follow-up was conducted eight months after surgery, at which time the wound was completely epithelialized, there was no gingival recession or irregular gingival morphology, and the gingival color was improved (Figure 4f). The postoperative OCT images showed no detectable white dots, which had been present preoperatively, suggesting that the metal fragments deposited in the connective tissue had been removed (Figure 4g). During follow-up, unfortunately root fractures were detected for both maxillary central incisors and they were extracted and implants were placed in a private clinic. At the 13-year follow-up of depigmentation, the peri-implant gingiva exhibited a stable, healthy color without any evidence of recurrent discoloration (Figure 4h).

## 4. Discussion

The present study investigated the potential clinical application of OCT combined with Er:YAG laser microsurgery for the treatment of gingival metal tattoos. In the ex vivo model, strong reflective signals observed on OCT corresponded to the locations of metal fragments identified histologically. In the clinical cases, OCT enabled visualization and localization of metal fragments and provided guidance for their removal using Er:YAG laser microsurgery. All three cases showed uneventful postoperative healing without postoperative pain, with favorable clinical and esthetic outcomes.

OCT is a device that uses light interference to delineate structures inside tissues and is currently being applied for clinical practice in ophthalmology, dermatology, and cardiology fields. Patients benefit substantially from the absence of radiation exposure, unlike CT imaging, and OCT imaging is safe even for children and pregnant women. Recent advances in OCT technology have expanded its applications in dentistry and periodontology, including three-dimensional imaging and OCT angiography for detailed assessment of periodontal morphology and microvascular changes [16]. OCT has also been increasingly investigated as a non-invasive imaging modality for oral mucosal diseases [21]. In the present study, highly reflective OCT signals corresponded to the location of metal fragments identified histologically in the ex vivo model, supporting the interpretation of similar OCT findings observed in the clinical cases. Although definitive diagnosis of a metal tattoo requires histopathological examination [22], biopsy is not routinely performed, and clinical diagnosis generally relies on medical and dental history and visual examination [23]. By enabling non-invasive visualization of findings consistent with metal fragments embedded in gingival tissue, OCT may provide additional information for the diagnosis of metal tattoos. However, the present study did not directly evaluate the differences in the OCT images among metal tattoos, melanoma, pigmented nevi, or other non-metallic pigmented lesions, and further comparative studies are needed to determine the utility of OCT for differential diagnosis.

In the present cases, the metal tattoos could be removed uneventfully with little postoperative pain by a minimally invasive technique using an Er:YAG laser during microsurgery. Periodontal tissue imaging using OCT enabled visualization of the location of the deposited metal debris in the connective tissue and provided guidance for selective removal of the pigmented tissue. In this study, postoperative OCT imaging was performed after completion of wound healing. As OCT imaging can be performed in real time, future studies may investigate its potential for intraoperative guidance during Er:YAG laser microsurgery. Real-time visualization of residual metal fragments could potentially facilitate more targeted tissue removal while minimizing unnecessary ablation of surrounding gingival tissue.

Several surgical approaches have been reported for the treatment of gingival metal tattoos, including excision or muco-abrasion combined with free gingival or connective tissue grafting [1,2,3,4,5,24]. A recent literature review reported favorable esthetic outcomes with graft-based approaches, while these procedures require additional surgical intervention and, in the case of autogenous grafting, a donor site [5]. Laser-based approaches have also been reported as less invasive alternatives [7]. In contrast, the OCT-guided Er:YAG laser microsurgical approach used in the present cases was intended to selectively identify and remove metal fragments while preserving the surrounding gingival tissue. However, because the present study did not include a control or comparative treatment group, no conclusions can be drawn regarding differences in healing, esthetic outcomes, or treatment efficacy compared with conventional surgical approaches. Nevertheless, the present findings suggest that the OCT-guided Er:YAG laser microsurgical approach may represent an alternative minimally invasive treatment option for gingival metal tattoos.

The use of Er:YAG and Nd:YAG lasers in minimally invasive periodontal surgery has also been reported, supporting their potential application in tissue-preserving surgical approaches [25]. In this report, the Er:YAG laser was used under microscopic guidance to selectively ablate pigmented connective tissue with metal debris. Clinical improvement in gingival coloration was observed in all three cases, and all three patients were satisfied with the results. The treatment was divided into two to three sessions, securing sufficient blood flow to the marginal gingiva in order to prevent the risk of impaired healing of the marginal gingiva as well as the subsequent gingival recession of gingival deformity. The chair time for each treatment was approximately 20 min, and it is considered that the patients’ stress was tolerable, even though the patient had to attend 4–5 times for examinations, treatments and observations. In addition, as in our previous report [7], postoperative pain was minimal in the present study. The VAS was used to objectively assess the patients’ pain, and all three patients reported that they had no pain at all from immediately after the operation until day 7.

In Case 2, as can be observed from the OCT images, the metal debris in the connective tissue seem to have been removed; however, the postoperative discoloration slightly remained at 10 months after treatment. One possible reason is that even if the metal debris themselves are removed, colored connective tissue due to deposition of metal ions may remain. During extended observation, however, the pigmentation gradually diminished without further intervention. This long-term improvement suggests that the darkened connective tissue containing residual metal ions was progressively replaced by normally colored tissue through natural connective tissue remodeling. Importantly, long-term follow-up of up to 13 years was available for two of the three cases. In Case 2, favorable gingival coloration was observed at 13 years without further intervention. In Case 3, although the treated teeth were subsequently extracted because of root fractures and replaced with implants, the peri-implant gingiva exhibited a stable, healthy appearance without recurrent discoloration at the 13-year follow-up. These observations provide long-term clinical information, although they should be interpreted cautiously given the limited number of cases and the subsequent implant treatment in Case 3.

This study has several limitations. First, both the ex vivo experiment and clinical case series included only three samples or patients, and the ex vivo experiment was not designed to establish the diagnostic accuracy of OCT. Second, the clinical case series did not include a control or comparative treatment group, and long-term follow-up was available for only two of the three cases. In addition, OCT images were not independently evaluated by a blinded examiner, and esthetic outcomes were assessed qualitatively without objective quantitative measures. The OCT systems used in this study also represent an earlier generation of OCT technology, and differences in imaging performance compared with currently available devices may limit the generalizability of the present findings. Therefore, the present findings should be considered preliminary, and larger comparative studies using current OCT systems are needed to further evaluate the diagnostic and clinical utility of this approach.

## 5. Conclusions

In this limited case series, OCT enabled non-invasive visualization and localization of metal fragments associated with gingival metal tattoos. The findings suggest that OCT-guided Er:YAG laser microsurgery may represent a feasible and minimally invasive approach for the treatment of gingival metal tattoos. Further studies with larger sample sizes and comparative designs are needed to evaluate its diagnostic utility, safety, and treatment efficacy.

## Figures and Tables

**Table 1:** Clinical characteristics, OCT findings, treatment, and outcomes of the three cases.

Characteristic	Case 1	Case 2	Case 3
Age, years	32	48	34
Sex	Female	Female	Female
Location and clinical extent of pigmentation	Narrow band along the marginal gingiva of the left maxillary central incisor	Broad, severe pigmentation involving the marginal gingiva of the right maxillary central incisor	Dark band along the marginal gingiva of the right maxillary central incisor
Preoperative OCT findings	White signal area with slightly indistinct borders in the connective tissue	Diffuse white signal areas scattered in the connective tissue	Well-defined white dots in the connective tissue
Treatment sessions, n	2	3	2
Postoperative pain, VAS	0 throughout days 0–7 after each treatment session	0 throughout days 0–7 after each treatment session	0 throughout days 0–7 after each treatment session
Postoperative healing	Complete epithelialization; no gingival deformity or recession	Complete epithelialization; slight gingival recession	Complete epithelialization; no gingival recession or irregular gingival morphology
Clinical follow-up	1.5 months	13 years	13 years
Final clinical and qualitative esthetic outcome	Improved gingival coloration without deformity or recession	Improved gingival coloration with slight residual discoloration at 10 months; healthy pink-colored gingiva without discoloration at 13 years	Improved gingival coloration at 8 months; stable healthy peri-implant gingiva without discoloration at 13 years

OCT, optical coherence tomography; VAS, visual analog scale. Esthetic outcomes were assessed qualitatively based on clinical examination and photographs; no validated esthetic scale or objective colorimetric assessment was performed.

## Data Availability

The data presented in this study are available on request from the corresponding author.

## Abbreviations

The following abbreviations are used in this manuscript:

OCT	Optical Coherence Tomography
CT	Computed Tomography
EL-MIKS	Er:YAG Laser Micro-Keyhole Surgery
VAS	Visual Analog Scale
